# Reclaimed water driven lettuce cultivation in a hydroponic system: the need of micropollutant removal by advanced wastewater treatment

**DOI:** 10.1007/s11356-021-14144-6

**Published:** 2021-05-04

**Authors:** Robert Kreuzig, Jaqueline Haller-Jans, Cornelia Bischoff, Johannes Leppin, Jörn Germer, Marius Mohr, Alexa Bliedung, Thomas Dockhorn

**Affiliations:** 1 Institute of Environmental and Sustainable Chemistry, Technische Universität Braunschweig, Hagenring 30, 38106 Braunschweig, Germany; 2 Hans-Ruthenberg-Institut, Universität Hohenheim, Garbenstraße 13, 70593 Stuttgart, Germany; 3 Bioprocess Engineering in Water Management and Circular Economy, Fraunhofer Institute for Interfacial Engineering and Biotechnology, Nobelstraße 12, 70569 Stuttgart, Germany; 4 Institute of Sanitary and Environmental Engineering, Technische Universität Braunschweig, Pockelsstraße 2a, 38106 Braunschweig, Germany

**Keywords:** Wastewater, Activated sludge treatment, Ozonation, Biological activated carbon filtration, Micropollutants, Hydroponic system, Lettuce cultivation

## Abstract

For a novel approach of resource-efficient water reuse, a municipal wastewater treatment plant was extended at pilot scale for advanced wastewater treatment, i.e., ozonation and biological activated carbon filtration, and a hydroponic system for reclaimed water driven lettuce cultivation. The treatment specific wastewater lines with the corresponding lettuce plants, differentiated into roots and shoots, were monitored for priority wastewater micropollutants, i.e., acesulfame (sweetener), caffeine (stimulant), carbamazepine, diclofenac, ibuprofen, sulfamethoxazole with acetyl-sulfamethoxazole (human pharmaceuticals), 1*H*-benzotriazole, and 4/5-methylbenzotriazole (industrial chemicals). As clearly demonstrated, conventional tertiary treatment could not efficiently clean up wastewater. Removal efficiencies ranged from 3% for carbamazepine to 100% for ibuprofen. The resulting pollution of the hydroponic water lines led to the accumulation of acesulfame, carbamazepine, and diclofenac in lettuce root systems at 32.0, 69.5, and 135 μg kg^−1^ and in the uptake of acesulfame and carbamazepine into lettuce shoots at 23.4 and 120 μg kg^−1^ dry weight, respectively. In contrast, both advanced treatment technologies when operating under optimized conditions achieved removal efficiencies of > 90% also for persistent micropollutants. Minimizing the pollution of reclaimed water thus met one relevant need for hydroponic lettuce cultivation.

**Graphical abstract:**

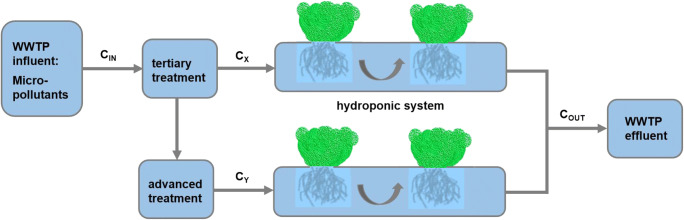

## Introduction

Wastewater, often polluted with household and industrial chemicals, needs efficient wastewater treatment in order to minimize the discharge load of pollutants from wastewater treatment plants (WWTP) into aquatic ecosystems. Since nearly 20 years, it has been well understood that micropollutants of different chemical classes, i.e., sweeteners, stimulants, human pharmaceuticals, X-ray contrast media, pesticides, and industrial chemicals, can only be partially removed via the conventional tertiary treatment technology (Pesqueira et al. 2020). Hence, WWTP effluents act as point sources for the entry of micropollutants into surface waters. As a consequence, advanced technologies have been under investigation since a long time, e.g., chlorination (Chamberlain and Adams 2006), UV irradiation (Adams et al. 2002; Russo et al. 2018), nanofiltration (Röhricht et al. 2009), ozonation, activated carbon filtration (Rattier et al. 2012; Völker et al. 2019), advanced oxidation processes (Kim et al. 2012; Margot et al. 2013; Reungoat et al. 2010, 2011; Sharma et al. 2018), and (bio)electrochemical technologies (Harnisch et al. 2013; Radenović et al. 2012). Until today, however, most of these advanced technologies could not been established area-wide in municipal WWTP (Ahting et al. 2018).

The removal of micropollutants from wastewater is of increasing environmental relevance going far beyond the protection of surface water quality. More than ever, the use of reclaimed water for irrigation farming is practiced in semiarid and arid regions where water scarcity is the most important natural constraint of economic growth and development (Al-Tarawneh et al. 2015; Wu et al. 2015). For this application pattern as well, it is important that irrigation water is not polluted to avoid soil pollution and, via plant uptake, the entry of micropollutants into the food chain. Particularly, this entry pathway was extensively studied showing that plant uptake depends on physicochemical properties of the micropollutants, habitus of the plants, soil properties, and irrigation water quality (Goldstein et al. 2014). These factors determine uptake, translocation, accumulation, and metabolism in plant organs. Riemenschneider et al. (2016) detected 18 of 34 organic micropollutants under study in field-grown vegetables, e.g., carrot, lettuce, potato, zucchini etc., irrigated with treated municipal wastewater, at concentrations from 1.7 to 216 μg kg^−1^ dry weight (dw). The micropollutants can be taken up via roots and shoots as proven in overhead- and surface-irrigation experiments (Bhlasod et al. 2018).

Alternatively to traditional soil-based plant cultivation, hydroponic systems supplied with solutions of micro- and macronutrients are used worldwide particularly for vegetable cultivation. This technology allows for the fine-tuning of water and nutrient supplies, improves plant productivity, avoids the need for crop rotation, and reduces pesticide application (Palermo et al. 2012). However, hydroponic systems are particularly sensitive to water pollution due to the lack of any buffer capacity as it is given in soil. Via direct root contact, pollutants may be taken up as shown in numerous studies (Carvalho et al. 2014; Herklotz et al. 2010).

In accordance with these aspects, the present study followed a novel approach of resource-efficient water reuse by downstreaming a hydroponic system for reclaimed water driven lettuce cultivation to a municipal wastewater treatment plant. The research objectives focused on (i) the analysis of selected micropollutants in municipal wastewater, (ii) the determination of the removal performance of conventional and advanced wastewater treatment technologies, and (iii) the impact of reclaimed water use on lettuce quality. For these purposes, samples of different wastewater lines were taken in 2017 and 2018 at a municipal WWTP additionally equipped for ozonation or biological activated carbon filtration (BACF) as 4th cleaning stages at pilot scale and analyzed applying liquid chromatography coupled to tandem mass spectrometry (LC/MS/MS). At the end of the respective vegetation periods, lettuce shoots and roots were sampled and analyzed to assess the impact of reclaimed water quality on the entry of micropollutants into lettuce shoots and thus into the food chain.

## Materials and methods

### Investigation site

The field study was performed at WWTP Wolfsburg-Hattorf, Germany, located at a rural and sparsely populated area gathering on average 396,000 m^3^ wastewater per year corresponding to a 6200 population equivalent (Bliedung et al. 2019, 2020). Here, a modular wastewater treatment system with different treatment technologies was installed to produce reclaimed water of different qualities for the use in hydroponic lettuce cultivation (*Lactuca sativa* L. var. Hawking RZ, Salanova; Rijk Zwaan Welver GmbH, Welver, Germany).

**Fig. 1:**
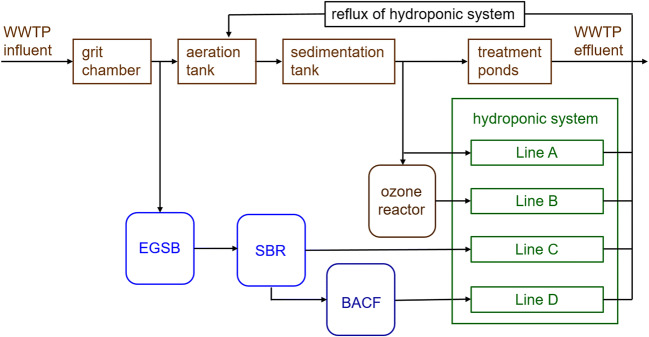
Wastewater lines from conventional and advanced wastewater treatment technologies to the hydroponic system for lettuce cultivation. WWTP: wastewater treatment plant, EGSB: expanded granular sludge bed reactor. SBR: sequencing batch reactor, BACF: biological activated carbon filtration reactor (adapted from Bliedung et al. 2020)

The basic wastewater treatment was performed, on the one hand, in the conventional WWTP (Fig. 1). The mechanical cleaning of debris and sand took place in a compact grit chamber. Subsequently, wastewater from this primary effluent was introduced into the aeration tank equipped with a biological phosphorus removal and an intermittent nitrification/denitrification unit supplied via tube aeration. Flocculants for phosphorous precipitation were added into the aeration tank. Then, wastewater was transferred to the secondary settling tank and finally to two tertiary treatment ponds before released into the outfall ditch.

On the other hand, as an alternative for low-capacity WWTP, the wastewater from the grit chamber was anaerobically treated first in an expanded granular sludge bed reactor (EGSB, volume, 0.3 m^3^; flow rate, 30–70 L h^−1^; hydraulic retention times, 4.5–10.5 h; ACS-Umwelttechnik GmbH Co. KG, Rielasingen-Worblingen, Germany). This process technology depletes organic substances without losses of ammonium and phosphorus compounds. Secondly, a sequencing batch reactor (SBR, volume, 0.96 m^3^; 1/3 volume of exchange; Technische Universität Braunschweig, Institute of Sanitary and Environmental Engineering, Braunschweig, Germany) operating under aerobic conditions was passed. Here, aerating and settling targeted at efficient nitrification. Denitrification and phosphorus removal, however, were dispensed with in order to reserve these macronutrients for reclaimed water use in the hydroponic system.

Advanced wastewater treatment technologies focused on ozonation and BACF. For ozonation as an already established technology (Luo et al. 2014; Margot et al. 2013; Pesqueira et al. 2020), wastewater from the secondary settling tank of the WWTP was transferred to an ozone reactor (volume, 0.30 m^3^; O_3_ dose, 8–9 g m^−3^; wastewater flow rate, 1.5 m^3^ h^−1^; Xylem Services GmbH, Herford, Germany). In contrast to practically relevant adsorption on powdered or granular activated carbon, a BACF reactor was applied at pilot scale (volume, 0.14 m^3^; flow rate, 30–60 L h^−1^; empty bed contact time, 2–4 h; Technische Universität Braunschweig, Institute of Sanitary and Environmental Engineering, Braunschweig, Germany). It was filled with 100 L granular activated carbon (Epibon A 4 × 8, 2.36–4.75 mm; Donau Carbon GmbH, Frankfurt, Germany) which had been exposed for 2 months before use to the WWTP effluent to cover the specific surfaces with biofilms allowing for sorption and biotransformation as simultaneously occurring processes for the removal of micropollutants (Rattier et al. 2012; Simpson 2008). During the experiments, the BACF system was flowed through with wastewater from the EGSB/SBR system at 30 L h^−1^ corresponding to 3000 bed volumes in the BACF reactor per year.

The hydroponic system was installed in a foil greenhouse allowing for air circulation at ambient temperature and natural sunlight illumination. It was operated as nutrient flow systems in PVC tubes (8 m length, 10 cm i.d.) with Line A, supplied with effluent from the secondary settling tank; Line B, supplied with effluent from the secondary settling tank after ozonation; Line C, supplied with the effluent of the EGSB/SBR system; and Line D, supplied with the effluent of the EGSB/SBR system after BACF (Fig. 1). A reference line was supplied with an adapted hydroponic nutrient solution (Epstein and Bloom 2005).

These tubes were installed without slope and equipped with drill holes for the lettuce plants (68 and 36 lettuce plants per tube in 2017 and 2018, respectively). Into these holes, 20-day-old seedlings were set. The tubes were filled with the reclaimed water to 2/3 for permanent rinsing of the lettuce roots. In 2017, the supply of the lettuce plants with reclaimed waters of different qualities was conducted in a flow-through mode, while in 2018 the reclaimed waters were periodically recycled for a more efficient exploitation of nutrients by the lettuce (feed & deplete mode; Bliedung et al. 2020). Surplus reclaimed waters were guided back into the WWTP aeration tank.

### Sampling activities

Sampling of treatment specific wastewater was conducted in 5 campaigns from September until November 2017 and in 6 campaigns from May until October 2018. Sampling positions were at the effluents of the grit chamber, secondary settling tank, ozone reactor, EGSB, SBR, BACF, and Line A to Line D of the hydroponic system. For analytical quality assurance, the influents were partly sampled as well. From the different wastewater lines, 10 random samples at minimum were taken per campaign assuming a homogeneous distribution of the micropollutants in wastewater due to efficient mixing and sufficiently long hydraulic retention times in the respective wastewater lines. In order to check for sampling quality, a 24-h composite sample was additionally taken during one day using an automatic sampler (Typ TPI, MAXX Mess- und Probenahmetechnik GmbH, Rangendingen, Germany) from the effluent of the grit chamber proving that both sampling activities led to matching results with differences of ± 24% for the target compounds. In order to check for the intraday variability of the wastewater pollution with the target compounds in the effluent of the grit chamber, 5 composite samples within 24 h were automatically taken and individually analyzed. A pollution evenly distributed was found. Average differences in the concentrations amounted for ± 31%.

During sampling at each wastewater line, 2-L wastewater samples were taken using a beaker and intermediately filled into 1-L ground-joint amber glass bottles. Directly thereafter, the samples were filtrated < 0.45 μm under low pressure (Pump SM 162/63/67, Sartorius, Göttingen, Germany) using glass fiber filters (MN 85/70 BF, Machery & Nagel, Düren, Germany) and filled into 1-L amber glass bottles which were transported in cooling boxes to the laboratory. There, the samples were stored at 4 °C for 3 days at maximum until analysis.

At harvest times in 2017 and 2018, respectively, 8 of 68 or 8 of 36 lettuce plants were sampled from the reference line and 4 of 68 or 4 of 36 ones from Line A to Line D of the hydroponic system corresponding with the respective wastewater treatment lines. The lettuce plants separated into shoots and roots were packed into freezer bags, transported in cooling boxes to the laboratory and stored there at − 20 °C until analysis.

### Wastewater analysis

According to Al-Tarawneh et al. (2015), the wastewater samples were analyzed for selected micropollutants often found in municipal wastewaters (Bahlmann et al. 2014; Castronovo et al. 2017; Jekel et al. 2015; Reemtsma et al. 2010; Ternes et al. 2007). Target compounds were acesulfame (sweetener), caffeine (stimulant), carbamazepine, diclofenac, ibuprofen, sulfamethoxazole and the corresponding metabolite acetyl-sulfamethoxazole (human pharmaceuticals), 1*H*-benzotriazole, and 4/5-methylbenzotriazole (industrial chemicals). Due to the analytical method applied, the methylbenzotriazole isomers are not distinguishable neither by retention times nor by mass transitions. Therefore, both are considered as a pair of target compounds (Riemenschneider et al. 2016). From these reference chemicals purchased from Dr. Ehrenstorfer (Augsburg, Germany), Sigma-Aldrich (Steinheim, Germany), HPC Standard GmbH (Cunnersdorf, Germany), or Riedel-de Haën (Seelze, Germany), single stock and mixed working standard solutions were prepared in methanol or acetonitrile (both LC grade, VWR Chemicals, Fontenay-sous-Bois, France) and stored at − 20 °C.

The 250-mL aliquots of the wastewater samples were transferred into 250-mL narrow mouth bottles. Formic acid (MS grade, Sigma-Aldrich, Steinheim, Germany) was added to adjust pH 4. Then, carbamazepine-d_10_ (10 μL from 10 ng/μL, HPC, Cunnersdorf, Germany) was spiked as the surrogate standard in order to check for losses during the sample preparation procedure. Across all wastewater samples, recoveries reached 97 ± 16%, revealing high quality of this sample preparation procedure. For solid-phase extraction (SPE), hydrophilic/lipophilic-balance cartridges (HLB, 500 mg, Waters, Eschborn, Germany) were applied. After conditioning the HLB cartridges with 5-mL methanol and 10-mL demineralized water acidified to pH 4 with formic acid, the wastewater samples percolated through at a flow rate of 3–4 mL min^−1^. After rinsing and 15-min low-pressure drying of the HLB cartridges, elution with 3 × 4 mL methanol followed. The pooled extracts were evaporated to < 0.5 mL in a gentle stream of nitrogen. The residues were reconstituted in 1 mL water/acetonitrile (50/50) with 0.1% formic acid. These analytical solutions were microfiltrated (0.2 μm, Chromafil PET-20/25, Machery & Nagel, Düren, Germany) into amber glass vials and stored at − 20 °C until analysis.

LC/MS/MS analysis was performed using a LC 1200 SL Series with degasser, binary pump, autosampler, and column oven (Agilent Technologies, Waldbronn, Germany) coupled to a 4000 QTrap tandem mass spectrometer (AB Sciex, Darmstadt, Germany) equipped with an electrospray ionization source (ESI, +/−). Chromatographic separation was achieved using a Zorbax Eclipse Plus C_18_ column (100 mm, 2.1 mm, 1.8 μm, guard column: 5 mm; Agilent Technologies, Waldbronn, Germany) at 20 °C. Eluents were A: water/acetonitrile (90/10) and B: acetonitrile/methanol (1/1), both added with 0.01% formic acid. The LC gradient program was time [min] / B [%]: 0/10, 15/100, 16/10, 28/10. Injection volume was 3 μL. Flow rate was 400 μL min^−1^. Target compound analysis was performed in multiple reaction monitoring (MRM) mode. Besides the retention times, identification focused on target compound specific mass transitions from precursor to 2 product ions to reach 4 identification points (EC 2002). In order to compensate matrix effects caused by different treatment specific wastewater qualities, single-point standard addition was consequently applied for quantitation. Furthermore, the quantifier/qualifier ion ratios (± 20%) and signal/noise ratios (S/N_QUAN_, ≥ 10; S/N_QUAL_, ≥ 3) were considered relevant for every target compound in every analytical run.

For analytical quality assurance, fortification experiments were conducted. Due to the lack of target compound free wastewater samples (zero samples), the method quantitation limits (MQL) were determined in tap water fully aware of the possible impact of matrix effects particularly on lowest determinable concentrations of the target compounds in real wastewater samples. Thus, highest matrix effects are expected particularly in the high-matrix loaded samples of the grit chamber effluent where, however, the target compounds occurred at concentrations definitively above MQL. These MQL reached from 0.002 μg L^−1^ for carbamazepine to 0.2 μg L^−1^ water for ibuprofen (Table 1).

**Table 1:** Average concentrations of selected micropollutants in wastewater before/after biological treatment in the aeration tank and in the hydroponic system Line A operated in flow-through mode in 2017

Target compound	ACE	CAF	CBZ	DIC	IBU	SMX	ASMX	BTZ	MBT
Conventional treatment									
Effluent grit chamber	14.1	110	1.56	7.09	19.0	0.59	1.56	13.1	1.42
*References: conc. max.*	*80* ^*1*^	*209* ^2^	*3.78* ^2^	*4.20* ^2^	*22.7* ^3^	*0.98* ^2^	*1.73* ^4^	*12.5* ^5^	*8.53* ^5^
Effluent secondary settling tank	0.76	0.09	1.52	3.20	< MQL	0.23	0.30	5.35	0.84
*References: conc. max.*	*2500* ^4^	*12.0* ^3^	*4.61* ^4^	*2.45* ^3^	*12.6* ^3^	*4.7* ^6^	*---*	*221* ^4^	*24.3* ^4^
Removal [%]	95	100	3	55	100	57	81	59	41
MQL water	0.002	0.01	0.002	0.002	0.20	0.01	0.01	0.20	0.01
Hydroponic system, Line A: reclaimed water from secondary settling tank									
Influent	0.51	0.12	1.49	2.28	< MQL	0.27	0.18	4.30	0.69
Effluent	0.60	0.17	1.56	2.42	< MQL	0.35	0.17	4.46	0.25
Lettuce shoots	23.4	< MQL	120	< MQL	< MQL	< MQL	< MQL	< MQL	< MQL
Lettuce roots	32.0	< MQL	69.5	135	< MQL	< MQL	< MQL	116	< MQL
MQL lettuce shoots	20	50	5	5	500	50	20	50	200
MQL lettuce roots	5	50	5	5	500	200	20	50	200

conc. max.: maximum concentrations. Concentration in wastewater: μg L^−1^. Concentration in lettuce: μg kg^−1^ dry weight. MQL: method quantitation limit

*ACE* acesulfame, *CAF* caffeine, *CBZ* carbamazepine, *DIC* diclofenac, *IBU* ibuprofen, *SMX* sulfamethoxazole, *ASMX* acetyl-sulfamethoxazole, *BTZ* 1*H*-benzotriazole, *MBT* 4/5-methylbenzotriazole

^1^Castronovo et al. (2017), ^2^Luo et al. (2014), ^3^Deblonde et al. (2014), ^4^Loos et al. (2013), ^5^Margot et al. (2013), ^6^García-Galán et al. (2016)

### Lettuce analysis

According to Riemenschneider et al. (2016), target compound analysis was performed for lettuce shoot and root samples. The unwashed samples were treated with liquid nitrogen, crushed with scissors and subsequently lyophilized (Alpha 1-2 LDplus, Christ, Osterode, Germany). Liquid nitrogen treated again, lyophilized shoot and root samples were pestled. The 0.5-g aliquots were transferred into centrifugation vials and spiked with the surrogate standard carbamazepine-d_10_. Across all lettuce samples, recoveries reached 101 ± 8% revealing high quality of the sample preparation procedure. After addition of 10-mL methanol, the samples were extracted on a horizontal shaker (KS 10 Digi, Bühler, Bodelshausen, Germany) at 300 rpm for 30 min followed by an ultrasound assisted extraction (Sonorex TK 52, Allpax, Papenburg, Germany) for 15 min. Thereafter, the suspensions were centrifuged (Megafuge 16R, Thermo Scientific, Dreieich, Germany) at 4000 g for 15 min. The supernatants were sequently microfiltrated at < 0.45 μm and < 0.20 μm using syringe filter units (20-mL disposable syringes; B. Braun Melsungen AG, Melsungen, Germany, Chromafil PET-45/25, Machery & Nagel, Düren, Germany).

Finally, the analytical solutions were LC/MS/MS analyzed as already described for wastewater analysis. As achieved in fortification experiments with lettuce shoot and root samples from the non-polluted reference line, MQL reached from 5 μg kg^−1^ dw for carbamazepine to 500 μg kg^−1^ dw for ibuprofen (Table 1).

## Results and discussion

### Removal of micropollutants via conventional wastewater treatment

The analyses of the grit chamber effluent of the sampling campaigns in 2017 clearly showed that the target compounds were detectable in every sample at μg L^−1^ concentrations. Highest concentrations were found for the stimulant caffeine at 110 ± 17.6 μg L^−1^, the human pharmaceutical ibuprofen at 19.0 ± 8.54 μg L^−1^, and the industrial chemical 1*H*-benzotriazole at 13.1 ± 4.64 μg L^−1^ wastewater. In 2018, matching data were found at 141 ± 36.3 μg caffeine L^−1^, 21.0 ± 8.62 μg ibuprofen L^−1^, and 11.8 ± 1.49 μg 1*H*-benzotriazole L^−1^. This wastewater pollution was well reflected by data reviewed for numerous other studies cited in Table 1 where, for the sake of clarity, only average concentrations are given clearly showing the performance of the wastewater treatment with direct impact on the pollution in the hydroponic system.

The concentration profiles from the effluent of the grit chamber to the effluent of the secondary settling tank confirmed that the micropollutants under study could only be partially removed via the conventional WWTP technology as also reported by Loos et al. (2013). Although acesulfame, caffeine and ibuprofen were removed by more than 95%, carbamazepine was to be classified non-removable from wastewater. For the other target compounds, removal rates ranged from 41% for 4/5-methylbenzotriazole up to 81% for acetyl-sulfamethoxazole (Table 1).

This reclaimed water was directly transferred to Line A of the downstream hydroponic system for lettuce cultivation operated in flow-through mode in 2017. Highest concentrations in the influent of this hydroponic system were found for carbamazepine, diclofenac, and 1*H*-benzotriazole at 1.49 ± 0.17, 2.28 ± 0.56, and 4.30 ± 0.72 μg L^−1^, respectively. The simultaneous analyses of the effluent led to matching results, i.e., for carbamazepine, diclofenac, and 1*H*-benzotriazole at 1.56 ± 0.14, 2.42 ± 0.37, and 4.46 ± 0.17 μg L^−1^, respectively, confirming the analytical quality from independent sampling to sample analysis.

Due to the permanent exposure of lettuce plants to micropollutants, residues in the lettuce root systems were found for acesulfame, carbamazepine, diclofenac, and 1*H*-benzotriazole at 32.0 ± 3.81, 69.5 ± 11.0, 135 ± 19.0, 116 ± 30.2 μg kg^−1^ dw roots. Since root samples were analyzed unwashed, a differentiation of micropollutants sorbed onto outer root surfaces or taken up into the roots was not possible, but also not necessary. On the one hand, micropollutants irreversibly sorbed onto outer root surfaces cannot be released by washing and, therefore, are inevitably identified as taken up as well. On the other hand, this differentiation is not relevant for the assessment of lettuce quality as long as only the lettuce shoots are yielded as cash crops.

In the lettuce shoots, acesulfame and carbamazepine were found at 23.4 ± 2.98 and 120 ± 8.76 μg kg^−1^ dw (Table 1). Particularly, the uptake of carbamazepine, also found in lettuce cultivated in soil at highest concentrations up to 345 μg kg^−1^ dw (Bhlasod et al. 2018), is to be regarded critical because its corresponding metabolite 10,11-epoxicarbamazepine, already detected in wastewater and plants, is assessed genotoxic (Riemenschneider et al. 2016). In contrast, lettuce roots and shoots from the reference line were free of residues.

Plant uptake and translocation within plants are attributed to physico-chemical properties of the micropollutants, particularly to lipophilicity and charge (Goldstein et al. 2014; Yahyazadeh et al. 2017). The less polar carbamazepine (LH_2_O: 0.15 g L^−1^; log K_ow_: 2.45; pK_A_: 13.9) (all data from PubChem, accessed on 03/30/2021) is taken up by the lettuce root hairs and is passed through root epidermis, cortex, and endodermis with Casparian strip due to its nonionic character in a wide pH range (Goldstein et al. 2018). Thus, it can easily cross selectively permeable plasma membranes and is subsequently translocated through the xylem into the lettuce shoots (Herklotz et al. 2010; Li et al. 2018). Such a transfer resulting in the accumulation of carbamazepine leaves and stems of peas was also reported by Tanoue et al. (2012).

Besides the root/shoot translocation of intermediately lipophilic compounds, a translocation of highly water-soluble, polar, nonionizable compounds was also reported by Dettenmaier et al. (2009). Even though ionizable, acesulfame (LH_2_O: 270 g L^−1^; log K_ow_: − 1.33; pK_A_: 2.0) was detectable in lettuce roots and shoots. Due to its high water solubility, the equilibrium concentration of its nonionic species at pH 7.0–7.4 of the cytosol in lettuce root cells is obviously high enough to ensure the membrane permeability demanded for the entry into the xylem. Such a translocation was experimentally proven for different soil-cultivated vegetables irrigated with reclaimed water by Riemenschneider et al. (2016). They also explained acesulfame concentrations of 21.7 μg kg^−1^ dw in carrot roots and 186 μg kg^−1^ dw in leaves with the translocation of acesulfame in neutral form.

In contrast, diclofenac (LH_2_O: 0.0024 g L^−1^; log K_ow_: 4.51; pK_A_: 4.15) dominantly occurs in reclaimed water (pH ≥ 7) in anionic form. Hence, its lowered lipophilicity might cause a reduced permeability through lipophilic, negatively charged membranes. Diclofenac seems to be thus rather ion trapped in the roots than translocated into the shoots (Tanoue et al. 2012). Zhai et al. (2016) thus determined bioaccumulation factors of 21 for roots and only of 0.16 for leaves of *Cyperus alternifolius.* Bartha et al. (2014) interpreted ten-fold higher diclofenac concentrations in roots than in shoots of *Typha latifolia* as a moderate translocation to upper plant parts due to a lower metabolic activity in leaves. Besides a rapid plant uptake, they observed an effective metabolism resulting in glycoside and glutathione conjugates of diclofenac as dominant metabolites.

1*H*-benzotriazole (LH_2_O, 1–5 g L^−1^; log K_ow_, 1.44; pK_A_, 8.37) was found in the lettuce root samples. Yet, it cannot be excluded that it just sticks to the cell wall and is not taken up into the root cells. This assumption is underlined by the finding that it was not detectable in the shoots even though its physicochemical properties range in-between those of carbamazepine and acesulfame. Alternatively, 1*H*-benzotriazole might be modified directly after uptake, as it is reported for the phytotransformation of benzotriazoles in sunflowers (Castro et al. 2003). LeFevre et al. (2015) identified the tryptophan biosynthesis and the glycosylation as rapid biotransformation pathways in *Arabidopsis* plants. They finally found that glycosylated 1*H*-benzotriazole was excreted by the plants into the hydroponic medium.

### Removal of micropollutants via conventional wastewater treatment and ozonation

The integration of the ozone reactor as 4th cleaning stage before Line B of the hydroponic system considerably increased the removal efficiency for the target compounds in 2017 even though the ozone dosage varied during this project period because of several failures of the feed pump supplying the ozone reactor (Table 2).

**Table 2:** Average concentrations of selected micropollutants in wastewater before/after conventional treatment in the aeration tank followed by ozonation as well as in the hydroponic system Line B operated in flow-through mode in 2017

Target compound	ACE	CAF	CBZ	DIC	IBU	SMX	ASMX	BTZ	MBT
Conventional treatment									
Effluent grit chamber	14.1	110	1.56	7.09	19.0	0.59	1.56	13.1	1.42
Effluent secondary settling tank	0.76	0.09	1.52	3.20	< MQL	0.23	0.30	5.35	0.84
Removal [%]	95	100	3	55	100	57	81	59	41
Ozonation									
Effluent ozone reactor	0.35	0.06	0.13	0.21	< MQL	0.07	0.10	2.23	0.23
Removal [%]	98	100	92	97	100	88	94	83	84
Hydroponic system, Line B: reclaimed water from ozone reactor									
Influent	0.37	0.11	0.02	0.17	< MQL	0.10	0.12	1.99	0.20
Effluent	0.48	0.06	< MQL	0.09	< MQL	0.08	0.09	1.94	0.36
Lettuce shoots	< MQL	< MQL	12.2	< MQL	< MQL	< MQL	< MQL	< MQL	< MQL
Lettuce roots	26.5	< MQL	12.4	12.2	< MQL	< MQL	< MQL	< MQL	< MQL

Concentration in wastewater, reclaimed water: μg L^−1^. Concentration in lettuce: μg kg^−1^ dry weight. MQL: method quantitation limit

*ACE* acesulfame, *CAF* caffeine, *CBZ* carbamazepine, *DIC* diclofenac, *IBU* ibuprofen, *SMX* sulfamethoxazole, *ASMX* acetyl-sulfamethoxazole, *BTZ* 1*H*-benzotriazole, *MBT* 4/5-methylbenzotriazole

Nevertheless, carbamazepine, diclofenac, sulfamethoxazole, and acetyl-sulfamethoxazole were removed by 92%, 97%, 88%, and 94%, respectively, resulting in low concentrations in the effluent of the ozone reactor as well as in influent and effluent of the hydroponic system. Likewise low concentrations were thus found in the lettuce root systems.

A higher persistence against ozonation was revealed for 1*H*-benzotriazole and 4/5-methylbenzotriazole by removal rates of only 83% and 84%. Respective removal tendencies were also reported by Altmann et al. (2014). Hence, residues in the effluent of the ozone reactor amounted to 2.23 ± 0.73 μg L^−1^ for 1*H*-benzotriazole and 0.23 ± 0.20 μg L^−1^ for 4/5-methylbenzotriazole. Matching data were determined in the corresponding influent and effluent samples of Line B of the hydroponic system. In contrast particularly to acesulfame, carbamazepine, and diclofenac, here, the benzotriazoles did not indicate any accumulation in lettuce roots or shoots. The same situation was found by Riemenschneider et al. (2016) analyzing lettuce which was soil-based cultivated and irrigated with surface water polluted with 1*H*-benzotriazole and 4/5-methylbenzotriazole at an average concentration of 0.4 μg L^−1^.

For the first sampling campaigns from May to July 2018, a constant ozone dosage could be achieved increasing the removal performance for the target compounds. Under these optimized conditions, even 1*H*-benzotriazole was removed by 93%. How sensitive lettuce plants can respond in complex wastewater treatment and lettuce cultivation experiments was shown after the failure of the feed pump supplying the ozone reactor in August 2018. Directly thereafter, particularly carbamazepine and diclofenac could not be efficiently removed resulting in concentrations high enough for accumulation on/in the lettuce roots, i.e., 55.8 μg carbamazepine and 27.4 μg diclofenac kg^−1^ dw. Carbamazepine was translocated again into the shoots at 104 μg kg^−1^ dw. In the samples taken during the following campaign in October 2018, carbamazepine was still found at 25.4 μg kg^−1^ dw roots and at 28.2 μg kg^−1^ dw shoots. Diclofenac was only found on/in lettuce roots at 26.0 μg kg^−1^ dw.

### Removal of micropollutants via EGSB/SBR system

Alternatively to the aeration tank, the EGSB/SBR system was tested at pilot scale as an anaerobic/aerobic wastewater treatment technology applicable for low-capacity WWTP. Compared to the removal performance for micropollutants of the conventional tertiary wastewater treatment, however, a clear inferiority was particularly found for the EGSB operating under anaerobic conditions. There even ibuprofen readily biodegradable in the aeration tank (removal: 100%) behaved persistent (Table 3). Despite the downstream aerobic SBR treatment, ibuprofen was removed only to a total of 87%. Besides the partial removal of the other target compounds, carbamazepine and 1*H*-benzotriazole were non-removable clearly demonstrating the demand of an advanced wastewater treatment technology.

**Table 3:** Average concentrations of selected micropollutants in wastewater before/after biological treatment in the EGSB/SBR system followed by BACF as well as in the hydroponic system Line C and D operated in flow-through mode in 2017 and recycling mode in 2018

Target compound	ACE	CAF	CBZ	DIC	IBU	SMX	ASMX	BTZ	MBT
Conventional treatment									
Effluent grit chamber	9.42	127	1.23	6.14	20.0	0.54	1.36	12.4	1.93
EGSB/SBR system									
Effluent ESGB	14.5	27.6	1.19	3.85	20.8	0.93	0.36	17.8	2.19
Removal [%]	0	78	3	37	0	0	74	0	0
Effluent SBR	2.59	10.0	1.39	2.32	2.66	0.17	0.32	14.4	1.70
Removal [%]	73	92	0	62	87	69	77	0	12
Hydroponic system, Line C: reclaimed water from EGBR/SBR system									
Reclaimed water	1.04	1.51	1.51	2.14	0.30	0.23	0.09	8.62	1.12
Lettuce shoots	92.5	< MQL	127	< MQL	< MQL	< MQL	< MQL	< MQL	< MQL
Lettuce roots	99.3	80.9	65.1	180	< MQL	< MQL	< MQL	525	< MQL
EGSB/SBR/BACF system									
Effluent BACF	0.21	0.07	0.03	0.03	< MQL	0.02	0.06	0.86	0.18
Removal [%]	98	100	98	100	100	96	96	93	91
Hydroponic system, Line D: reclaimed water from EGBR/SBR/BACF system									
Reclaimed water	0.22	0.02	0.08	0.05	< MQL	0.02	0.01	1.06	0.32
Lettuce shoots	< MQL	< MQL	7.35	< MQL	< MQL	< MQL	< MQL	< MQL	< MQL
Lettuce roots	31.4	< MQL	126	158	< MQL	< MQL	< MQL	432	228

*EGSB* expanded granular sludge bed reactor, *SBR* sequencing batch reactor, *BACF* biologically activated carbon filtration. Concentration in wastewater, reclaimed water: μg L^−1^. Concentration in lettuce: μg kg^−1^ dry weight. *MQL* method quantitation limit, *ACE* acesulfame, *CAF* caffeine, *CBZ* carbamazepine, *DIC* diclofenac, *IBU* ibuprofen, *SMX* sulfamethoxazole, *ASMX* acetyl-sulfamethoxazole, *BTZ* 1*H*-benzotriazole, *MBT* 4/5-methylbenzotriazole

Due to the limited removal performance of the EGSB/SBR system, the reclaimed water use in Line C of the hydroponic system led to a relevant accumulation of the target compounds on/in the lettuce roots. Particularly high concentrations were found for diclofenac and 1*H*-benzotriazole at 180 ± 121 and 525 ± 207 μg kg^−1^ dw, respectively, mainly caused in 2017 when the reclaimed water regime was operated in flow-through mode. However, these findings were not reflected by different concentrations in the reclaimed water in 2017 and 2018 which only slightly varied for diclofenac at 2.14 ± 0.84 μg L^−1^ and for 1*H*-benzotriazole at 8.62 ± 3.62 μg L^−1^. In the lettuce shoots again, only acesulfame and carbamazepine were detected at 92.5 ± 69.4 and 127 ± 55.6 μg kg^−1^ dw.

### Removal of micropollutants via EGSB/SBR/BACF system

In order to enhance the removal performance of the EGSB/SBR system, a BACF reactor was connected downstream as a well-proven technology for high removal performance for different micropollutants from wastewater (Reungoat et al. 2010; Margot et al. 2013). Thus, the target compounds were removed by more than 91% (Table 3). In comparison to ozonation, the removal rates of carbamazepine, 1*H*-benzotriazole, and 4/5-methylbenzotriazole increased from 92 to 98%, 83 to 93%, and 84 to 91%, respectively. Due to sorption and biotransformation as simultaneously occurring processes in the BACF reactor, this system has remained its removal performance since the beginning of these investigations in 2017.

This combination of conventional and advanced wastewater treatment technologies led to a lower reclaimed water pollution in Line D of the hydroponic system. Contradictory to this, however, the high concentrations of acesulfame, carbamazepine, diclofenac, 1*H*-benzotriazole, and 4/5-methylbenzotriazole on/in the lettuce roots were particularly noticeable. These findings might be caused during the reclaimed water regime in flow-through mode in 2017 linked to a temporary release of activated carbon particles out of the BACF reactor. These particles loaded with micropollutants were enriched in the lettuce root systems and were co-extracted together with the unwashed root samples during the sample preparation procedure. The extracted target compounds were finally detected in the analytical solutions via LC/MS/MS. In contrast, the lettuce roots of the sampling campaigns in 2018 were free of residues.

In comparison to Line C, where the carbamazepine average concentration in roots of 65.1 μg kg^−1^ dw corresponded to 127 μg kg^−1^ dw shoots, the carbamazepine transfer in lettuce plants of Line D was limited. There, higher concentrations in roots at 126 μg kg^−1^ dw corresponded only to 7.35 μg kg^−1^ dw shoots. These findings indicated that carbamazepine sorbed onto activated carbon particles was only marginally bioavailable for lettuce. Apart from this system failure, the BACF system proved highest removal performance for micropollutants in effluents from conventional wastewater treatment technologies needed for high-quality lettuce cultivation in reclaimed water driven hydroponic systems.

## Conclusion

In this monitoring study, it was clearly demonstrated that wastewater can be a valuable water and nutrient resource for cash crop cultivation in hydroponic systems. This may be a substantial step in the investigation of alternative water resources for irrigation farming particularly in semiarid and arid regions. It was clearly demonstrated as well that the reclaimed water has to be largely free of micropollutants to avoid their plant uptake, bioaccumulation, and entry into the food chain. For this purpose, however, advanced wastewater technologies are demanded which are even not established area-wide in state-of-the-art municipale WWTP. As an appropriate treatment scheme, therefore, a combination of EGSB, SBR, and BACF may be advantageous due to energy efficient wastewater treatment, maintaining of the nutrients as well as an efficient removal of organic micropollutants. Taking into account that the quality of lettuce and vegetables, consumed fresh only after washing without any additional processing, may be interfered by micropollutants, but also by pathogenic and/or resistant bacteria, ozonation may be the more efficient advanced wastewater technology destroying micropollutants and controlling bacteria. For a final assessment of reclaimed water driven hydroponic systems for cash crop cultivation, thus, additional information by upscaling the experiments considering other wastewater qualities, treatment technologies, test plants, etc. is necessary.

## Data Availability

All data generated or analyzed during this study are included in this published article.
